# The development and effects of a social constructivist approach in an interprofessional discomfort care online education program

**DOI:** 10.1186/s12909-024-06342-w

**Published:** 2024-11-26

**Authors:** Young-Rim Choi, Ye-NA Lee, Dai Young Kwon, Dayeong Kim, Won Hee Park, Sung Ok Chang

**Affiliations:** 1 Department of Nursing, Far East University, Eumseong-Gun, Chungcheongbuk-Do, 27601 Republic of Korea; 2 Department of Nursing, The University of Suwon, Hwaseong, Gyeonggi-do 18323 Republic of Korea; 3 Gifted Education Center, Korea University, Seoul, 02841 Republic of Korea; 4 College of Nursing and BK21 FOUR R&E Center for Learning Health Systems, Korea University, Seoul, 02841 Republic of Korea; 5 College of Nursing, Korea University, Seoul, Republic of Korea

**Keywords:** Long-term care facility, Discomfort, Dementia, Interprofessional education, Social constructivist, Online education

## Abstract

**Background:**

The importance of interprofessional education (IPE) programs is increasing due to the complexity and multidimensional aspects of discomfort in long-term care facilities (LTCFs). The social constructivist approach, which is helpful in IPE, has received considerable attention in education. This study aimed to develop and identify the effects of an interprofessional discomfort management online education program using a social constructivist approach.

**Methods:**

Using the Network-Based Instructional System Design model, five steps were employed for online educational program development: analysis, design, production, implementation, and evaluation. We modified the framework of interprofessional discomfort care and a C3 (case-based, collaborative, and contextual learning) instructional model to construct the program. The study used a non-equivalent control group pre-post-test design with 54 interprofessional participants from four LTCFs.

**Results:**

The pre-post outcomes were statistically significant for proactivity in problem-solving (t = − 2.244, *p* = 0.030), team outcomes (t = − 2.457, *p* = 0.017), and transactive memory system (t = − 3.229, *p* = 0.002). The results of the learners’ educational program-related satisfaction were as follows: overall degree of satisfaction, 3.67 ± 0.67; difficulty, 3.56 ± 0.82; suitability for practice, 3.83 ± 0.64, content, 3.69 ± 0.75; and educational method, 3.46 ± 0.86.

**Conclusions:**

These findings support the effectiveness of the social constructivist approach education program for the awareness of discomfort care in LTCF healthcare professionals and can contribute to the improvement of IPE.

## Background

The importance of interprofessional care (IC) in healthcare is gradually increasing. IC has been shown to provide high-quality care to patients and to improve their health outcomes [1, 2]. In long-term care facilities (LTCFs), IC is a critical concept. Staff in LTCFs, such as nurses, caregivers, social workers, physical therapists, and occupational therapists, cooperate to achieve the common goal of improving the safety and quality of life of residents in LTCFs [3]. In particular, an interprofessional approach is essential for managing discomfort in LTCFs.

Discomfort is the opposite of comfort and refers to a state of mental or physical unease [4]. It affects physical and psychological states, resulting in various symptoms (e.g., fatigue, sleeplessness, isolation, and embarrassment) [5]. Older adults with dementia, who account for 48.5% of the residents in LTCFs [6], have difficulty communicating because of their cognitive decline, which leads to difficulty in expressing the various symptoms of discomfort [7]. This causes practitioners to underestimate and inadequately manage discomfort [8]. Discomfort that is not effectively managed in LTCFs not only adversely affects the quality of life of residents with dementia but also threatens their physical and psychological safety [9].

Moreover, as discomfort comprises multidimensional aspects of physical, environmental, and social psychology, it is difficult for one practitioner to manage the complexity of discomfort, making it necessary for practitioners to implement IC [10]. Findings from previous studies have highlighted the importance of collaborative interprofessional education (IPE) programs that help to enable effective IC by enhancing the competency of practitioners [9, 11]. In particular, the social constructivist approach has proven to be an effective method of education for IPE [12, 13].

However, the COVID-19 pandemic posed a daunting challenge for the education system. In the context of the pandemic, the need for a new method of training differing from traditional learning trajectories, for example, face-to-face learning, for practitioners in LTCFs has emerged [14]. The growth of online learning, an education system transformed by the COVID-19 pandemic that is constrained by neither time nor space, has accelerated as its importance as an alternative learning method has become more recognized [15, 16].

### Overview of social constructivism

Constructivism is the epistemology of cognition in which knowledge is actively formed by the individual. This concept contradicts traditional epistemology, which holds that truth exists externally regardless of human experience [17, 18]. Constructivism can be classified as cognitive and social. If an individual’s cognitive structure plays an essential role in knowledge formation, it is called cognitive constructivism. Alternatively, social constructivists believe that social interactions are crucial to knowledge formation [19, 20].

In other words, the knowledge in social constructivism is formed through dialogue and interaction with others [21], which is why cooperation and dialogue are fundamental to education. Therefore, from the perspective of social constructivism, class design is pursued for maximizing the interaction between educators and learners, and between learners in the classroom. Accordingly, teachers and students must cultivate relationships in which they participate in dialogues and solve problems through practical and participatory activities [22].

Barak and Green (2020) [23] presented a C3 instructional model for this social-constructivist approach to education. In the C3 instructional model, online learning activities merge case-based, collaborative, and contextual learning. Through case-based learning, learners develop critical thinking and problem-solving skills; collaborative learning helps increase participation and promote academic achievement; and contextual learning encourages learners to participate in practical learning assignments [23].

This study aimed to develop a discomfort management IPE online program based on social constructivism and to confirm its effectiveness.

## Method

**Fig. 1:**
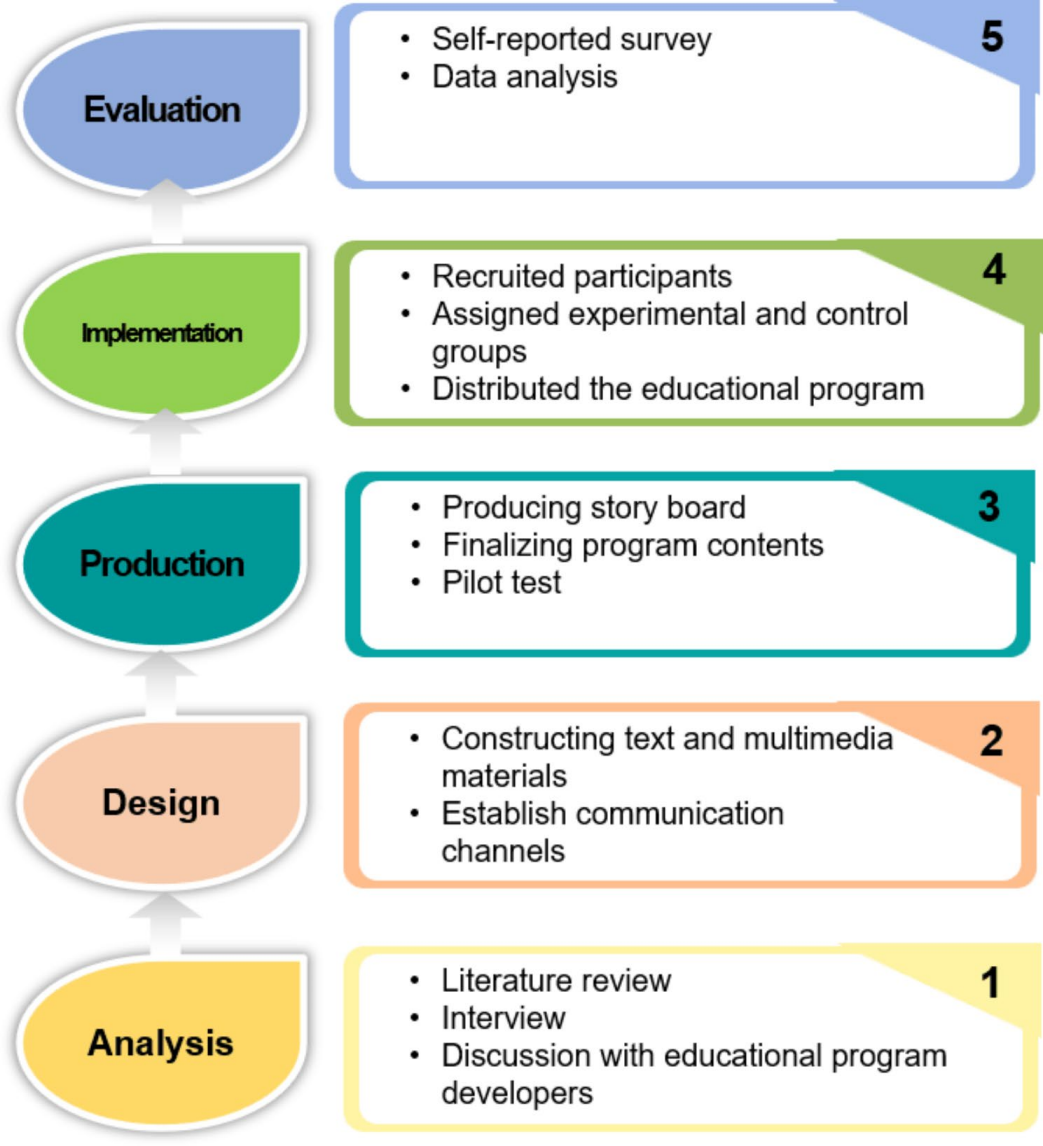
The layout for developing an online educational program adapting the Network-Based Instructional System Design model

The Network-Based Instructional System Design (NBISD), a stepwise approach, was employed [24]. The NBISD comprises five steps: analysis, design, production, implementation, and evaluation (Fig. 1).

### Step 1. Analysis

The first step was to identify the educational content and needs. To identify learners’ needs, this study interviewed 17 participants. During the interviews, we investigated cases of discomfort management and educational needs.

The average age of the participants was 46.5 (range 27–64) years, and their average work experience was 5.2 (range 3–10) years. Nine registered nurses, four social workers, three physical therapists, and one nurse assistant participated in the interviews, which lasted 60 min each. We used semi-structured questions concerning discomfort management cases, educational goals and content, media, and training time.

The findings of the interviews are as follows. The goals and contents of this program focused on understanding and assessing the concept of discomfort. Online learning was found to be a suitable medium for coping with the COVID-19 pandemic and the resulting increase in staff workload. The participants recommended a training time of 2–3 h as the appropriate educational duration. Educational program developers were hired to conduct an environmental analysis. Researchers and developers have several times discussed setting up online the educational program environment.

### Step 2. Design

Step 2 involved designing and materializing the outputs of Step 1. Text and multimedia materials were constructed to specify educational goals, content, methods, and strategies.

**Fig. 2:**
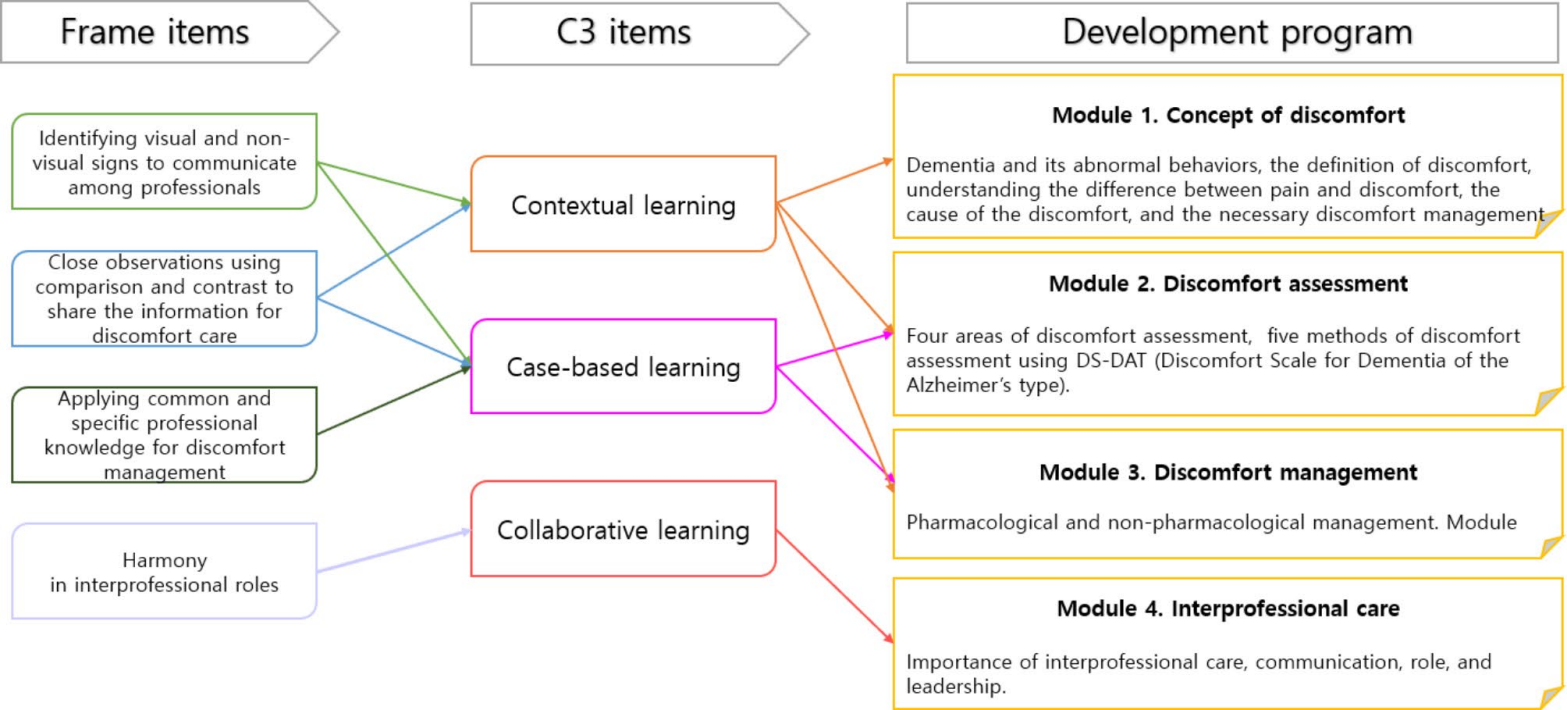
Designing education program adapted from both interprofessional discomfort management frame and C3 instructional model

We designed an educational program by combining a framework of interprofessional discomfort care developed by Choi et al. (2023) [25] and a C3 instructional model developed by Barak and Green (2020) [23] (Fig. 2). C3 helps apply the social constructivist perspective to education in online courses [23], and the framework informs the core elements of interprofessional discomfort management in LTCF as a basic outline for creating educational programs [25]. Therefore, we combined these two theoretical approaches to obtain new insights into real education for professional healthcare providers in LTCFs.

Choi et al.’s (2023) [25] framework comprises four concepts: (1) identifying visual and non-visual signs to be communicated among professionals; (2) close observations using comparison and contrast to glean and share information for discomfort care; (3) applying common and specific professional knowledge for discomfort management; and (4) harmony between interprofessional roles. Barak and Green (2020) [23] presented an instructional model for case-based, collaborative, and contextual learning in C3.

In the current study, discussions among learners and feedback on the opinions of other learners were provided for collaborative and harmonious learning among interprofessional practitioners, and corresponding interactions through educator–learner chats were designed. For case-based learning on identifying discomfort via close observation, the participants were provided a case in which they were to solve the problem of managing actual discomfort in a LTCF. We also provided related video material. An educational model was constructed to combine roles and knowledge in discomfort management from nursing, physics, and sociological perspectives for contextual learning and knowledge application.

### Step 3. Production

Step 3 involved the production of the educational content and system. An online program was developed for the study since online education can act as a bridge between increasing IPE needs and implementation in LTCF environments [26]. A storyboard based on the educational content in Step 2 was created by the first author. The program contents were finalized by revising and supplementing the educational content through discussions among researchers, and the program’s online format was optimized using the Google Chrome web browser. The educational program was pilot tested over two weeks to check for operational errors.

### Step 4. Implementation

In Step 4, we recruited the participants and conducted an educational program. Practitioners from four LTCFs were recruited for the study. The inclusion criterion was to be a practitioner currently working in an LTCF, while the exclusion criterion was difficulty using a computer program. G*Power 3.1.9.4 (Heinrich-Heine-Universität, Düsseldorf, Germany) was used to calculate the sample size. When the power was 0.8, the significance level was 0.05, and the effect size was 0.80 [27], both the experimental and control groups of this study were confirmed to have proper sample sizes.

A total of 58 people from four LTCFs in South Korea participated in the study; 54 completed the educational program. A non-equivalent control group pre-/post-test design was used. Of the 54 participants in this study, 28 were placed in the experimental group and 26 in the control group. The experimental group underwent a social constructivist approach-based educational program, whereas the control group took instruction from a conventional online program.

### Step 5. Evaluation

The last step analyzed satisfaction with the educational program and the learning effect. Data were obtained on the effectiveness of learning satisfaction and evaluation tools using a self-administered questionnaire. The outcomes of learners were measured using the Korean versions of “proactivity in problem-solving,” “team outcomes,” “transactive memory system,” and “education program-related satisfaction.”

#### Proactivity in problem-solving

“Proactivity in problem-solving” measured the awareness of finding alternatives to problems and actively solving them [28]. Each item was measured on a 5-point Likert scale ranging from 1 point for “not at all” to 5 points for “very much so,” with a high score indicating an increase in proactive problem-solving. The evaluation tool consisted of eight items and had internal reliability with a Cronbach’s alpha of 0.89.

#### Team outcomes

“Team outcomes” is an evaluation tool to show the team performance, commitment, and satisfaction of team members. Each item was measured on a 5-point Likert scale ranging from 1 point for “not at all” to 5 points for “very much so” [29]. Higher scores indicated higher team performance. The tool showed internal reliability, with a Cronbach’s alpha of 0.90, and consisted of five items.

#### Transactive memory system

The “transactive memory system (TMS)” refers to the cooperative division of labor for storing, retrieving, and communicating team knowledge that identifies team members’ knowledge and coordinates between different sources of knowledge to achieve a task that the entire team recognizes jointly [29, 30]. Therefore, information exchange systems contribute to team performance by inducing the integration of team members’ knowledge [31]. The measure consisted of expertise (five items), reliability (five items), and task coordination (five items). A 5-point Likert scale was used for each item ranging from 1 point for “not at all” to 5 points for “very much so.” In this study, the higher the score, the higher was the internal reliability of the transactive memory system, with a Cronbach’s alpha of 0.92.

#### Educational program-related satisfaction

Educational program-related satisfaction was assessed by the researcher of this study using five items that assessed the participants’ degree of satisfaction—difficulty, suitability for practice, content, education method, and overall satisfaction—after the online program intervention. Items were scored on a 5-point Likert scale ranging from 1, “not at all,” to 5, “very much;” a higher score indicated a higher degree of satisfaction. Cronbach’s alpha was 0.90 in this study.

#### Data analysis

IBM SPSS software (version 25.0) was used for all statistical analyses. Descriptive statistics were used to analyze the participants’ demographic information. A two-sample *t*-test was performed to confirm the differences in the outcome variables between the two groups before and after the program. Statistical analyses were performed at a significance level of 0.05.

#### Ethical considerations

This study was approved by the Korea University Institutional Review Board (Clinical Trial Number: KUIRB-2021-0222-01). All participants began the study after providing written consent. Participation was voluntary, and participants could withdraw from the study at any time.

## Results

### Development of an online educational program with a social constructivist approach

**Fig. 3:**
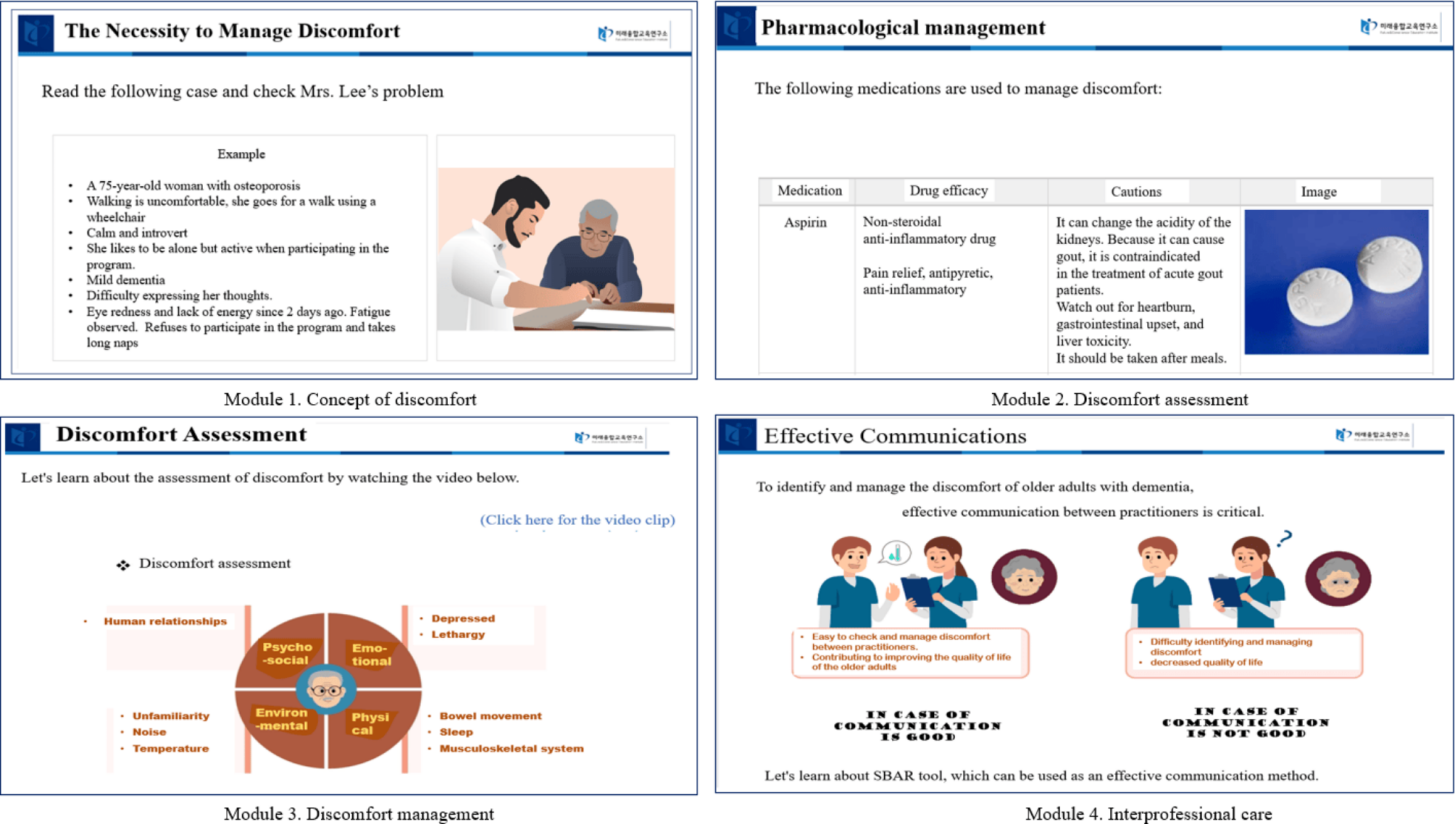
Example scenes of interprofessional discomfort management education program in long term care facilities

Figure 3 shows the modules and examples of the developed online educational program. Participants learned the concept, assessment, and management of discomfort according to the C3 instructional model and the interprofessional discomfort management framework. The four major modules of the program are: module (1) concept of discomfort: dementia and its related abnormal behaviors, definition of discomfort, understanding the difference between pain and discomfort, causes of discomfort, and necessary discomfort management; module (2) discomfort assessment: four areas of discomfort assessment and five methods of discomfort assessment using the Discomfort Scale for Dementia of the Alzheimer’s Type; module (3) discomfort management: pharmacological and non-pharmacological management; and module (4) importance of interprofessional care: communication, roles, and leadership.

### Effects of an online educational program with a social constructivist approach

Table 1 presents the general characteristics of the participants. No statistically significant demographic differences were observed between the experimental and control groups.

**Table 1:** General characteristics and homogeneity test between two groups (*n* = 54)

Characteristics	Category	Exp(*n* = 28)	Cont(*n* = 26)	X^2^ or t	*p*-value
		Mean ± SD or *N* (%)			
Age		51.04 ± 7.10	53.81 ± 10.72	1.128	0.264
Clinical experience in NH (years)		6.18 ± 3.67	4.55 ± 3.74	-1.615	0.112
Academic degree	< Baccalaureate	25 (89.3)	23 (88.5)	0.094	0.925
	<=Master’s	3 (10.7)	3 (11.5)		
Pretest score of variables	Proactivity in problem solving	31.93 ± 5.16	31.58 ± 3.36	-0.299	0.767
	Team outcomes	19.14 ± 2.56	19.69 ± 3.69	0.640	0.525
	Transactive memory system	59.29 ± 4.78	58.04 ± 4.84	-0.952	0.345

Note: Exp = Experimental group; Cont = Control group; SD = Standard deviation; LTCF = Long-term care facility

The results of the online educational program that enhanced proactivity in problem solving, team outcomes, and the transactive memory system are presented in Table 2.

**Table 2:** Comparison of outcome variables between two groups (*n* = 54)

Variable (no of items)		Baseline		Post	T	*p*-value
		**Mean ± SD**				
Proactivity in problem solving (8)	Exp (*n* = 28)	31.93 ± 5.16	34.32 ± 5.19		-2.244	0.030^a^
	Cont (*n* = 26)	31.58 ± 3.36	31.69 ± 3.36			
Team outcomes (5)	Exp (*n* = 28)	19.14 ± 2.56	21.79 ± 2.70		-2.457	0.017 ^a^
	Cont (*n* = 26)	19.69 ± 3.69	19.62 ± 3.74			
Transactive memory system (15)	Exp (*n* = 28)	59.29 ± 4.78	63.14 ± 4.83		-3.229	0.002 ^a^
	Cont (*n* = 26)	58.04 ± 4.84	58.73 ± 5.21			

Note: Exp = Experimental group; Cont = Control group; SD = Standard deviation

^a^ Significant at < 0.05

With respect to proactivity in problem-solving, the control group averaged 31.69 points and the experimental group averaged 34.32 points (*p* = 0.030), which shows a statistically significant improvement in the experimental group. In terms of team outcomes, the control group averaged 19.62 points and the experimental group 21.79 points (*p* = 0.017), showing a statistically significant improvement in the experimental group. In the transactive memory system, the control group averaged 58.73 points and the experimental group 63.14 points (*p* = 0.002), again demonstrating a statistically significant improvement in the experimental group.

The results of the learners’ educational program-related satisfaction were as follows: overall degree of satisfaction, 3.67 ± 0.67; difficulty, 3.56 ± 0.82; suitability for practice, 3.83 ± 0.64, content, 3.69 ± 0.75; and, educational method, 3.46 ± 0.86.

## Discussion

The social-constructivist approach to IPE is not new. Since the 1990s, research has suggested that social constructivism-based collaborative learning improves group performance [32], and even recently it has proven to be an effective learning method [33].

To the best of our knowledge, this is the first study to develop and implement a discomfort education program using a social constructivist approach. Despite numerous studies on discomfort, such as those on conceptual definitions [10] and measurement tools [34], training programs addressing the subject are lacking, making it challenging to train practitioners. This study confirmed that training practitioners to manage discomfort in the clinical field is effective. In addition, the program development process, which enhances practical applicability by investigating cases and educational needs through interviews and theories such as the interprofessional discomfort management framework and C3 instructional model, provides insight into the direction of future practitioner training program development. In future studies, it will be necessary to expand from LTCFs to an acute setting in the management of discomfort of patients with dementia. Furthermore, research is needed to move beyond discomfort management to create a home-like environment for residents’ comfort [35]. This can promote residents’ sense of security and improve their quality of life [36].

This study used an online learning program development process, the NBISD model, to develop educational programs. The COVID-19 pandemic has played a role in the advancement of web-based learning [37]. More helpfully, progressive information technology can be a vehicle for applying the social constructivist theory. Using such technology not only enables learning that transcends time and space limits but also provides a way to foster active learning and problem-solving [16].

We used the C3 instructional model as the instruction concept. This is a systematic approach through case studies; the provision of combined knowledge from an interprofessional perspective and interaction enhances collaboration [38]. Interactive education can be improved through interprofessional learning, and collaborative awareness can be enhanced among students [38]. This study shows that this ability can be enhanced by practitioners.

The experimental group showed significantly better results in active problem-solving. Proactivity in problem-solving indicates an active attitude toward performing team tasks. In this study, the experimental group improved for two reasons. First, the examples for “case-based learning” in the program were extracted from interviews with practitioners. This reflects that many practical situations and authentic scenarios are familiar in real-world clinical environments [39]. In addition, while discussing how to deal with a problematic situation with team learning, it was observed that team learning could not be completed without active participation. For improving learner participation in team learning, one effective method is providing responsibility. However, participation may be difficult if the learning methods are not understood. Nonetheless, those in an age group unfamiliar with web-based learning, or beginners, may participate in the class after they are provided sufficient pre-operation instructions and confirmation.

TMS and team outcomes led to significantly improved results in the experimental group. The results of this study are similar to those of Lee et al. (2020) [26], who found that interprofessional practitioners in LTCFs improved their scores while learning through simulation plus a case-based study. A previous study found that team members in intensive care units work together using TMSs and that successful functioning can be promoted by aligning care with patient goals and priorities [40].

This study has some limitations. The first is that the program was limited to participants from four LTCFs in South Korea. We conducted this study during the peak of the COVID-19 pandemic, when strict social distancing restrictions were being imposed. Moreover, 100% of the participants in this study were women. The possibility of differences in collaboration between men and women cannot be ruled out [41]. A wide range of participants is required for expanded interpretation and application of the study’s findings. Another limitation of the study is that it did not consider the participants’ educational background. Interprofessional practitioners working in long-term care facilities have diverse academic backgrounds. Exploring their prior knowledge and combining it with practical knowledge will result in better training. Therefore, we recommend that future research consider programs based on the educational background of the participants. This was a one-time training program, but long-term observations are required to confirm the continuous effects of the educational programs. In addition, it is necessary to regularly develop course education programs that keep up with the accumulated knowledge and changes in practice. Although the number of studies on professional collaboration in practical settings is increasing, more rigorous research is needed.

## Conclusion

This study was conducted to develop an interprofessional discomfort management education program using a social constructivist approach and to confirm its effectiveness. To develop the educational programs, the C3 instructional model and interprofessional discomfort management framework were used, and a step-by-step online educational program, the NBISD model, was applied. As a result, we identified that the experimental group, which completed the interprofessional discomfort online education program with a social constructivist approach, showed significant improvement in proactivity in problem-solving, team outcomes, and the TMS. This study reduces the gap between education and practice by collecting data from both theoretical and practical perspectives. Future studies should consider cooperative learning in various ways to break down professional barriers and better harmonize collaborative practices.

## Data Availability

Data collected during interviews is not available due to the sensitive nature of the information (such as personal details) contained therein, but other data is available upon reasonable request from the corresponding author.

## Abbreviations

IC: Interprofessional care
IPE: Interprofessional education
LTCFs: Long-term care facilities
NBISD: Network-Based Instructional System Design
TMS: Transactive memory system
